# The mechanism and therapeutic potential of lncRNA MIR497HG/miR-16-5p axis in breast cancer

**DOI:** 10.1186/s12905-024-03208-7

**Published:** 2024-07-02

**Authors:** Quan Cheng, Dong-Yang Yu, Yong-Hong Zhou, Jian-Yuan Huang

**Affiliations:** 1 Department of Chinese Medicine, Hangzhou First People’s Hospital, Hangzhou, 310006 China; 2 Department of Urology, People’s Hospital of Yilong County, Nanchong, 637600 China; 3 Department of General Surgery (Thyroid Gland/Blood Vessel), The First People’s Hospital of Neijiang, No. 1866, West Section of Han’an Avenue, Neijiang, 641099 China

**Keywords:** Breast cancer, lncRNA MIR497HG, miR-16-5p, Diagnosis, Migration, Invasion

## Abstract

**Background:**

Breast cancer has become a major public health problem in the current society, and its incidence rate ranks the first among Chinese female malignant tumors. This paper once again confirmed the efficacy of lncRNA in tumor regulation by introducing the mechanism of the diagnosis of breast cancer by the MIR497HG/miR-16-5p axis.

**Methods:**

The abnormal expression of MIR497HG in breast cancer was determined by RT-qPCR method, and the correlation between MIR497HG expression and clinicopathological characteristics of breast cancer patients was analyzed via Chi-square test. To understand the diagnostic potential of MIR497HG in breast cancer by drawing the receiver operating characteristic curve (ROC). The overexpressed MIR497HG (pcDNA3.1-MIR497HG) was designed and constructed to explore the regulation of elevated MIR497HG on biological function of BT549 and Hs 578T cells through Transwell assays. Additionally, the luciferase gene reporter assay and Pearson analysis evaluated the targeting relationship of MIR497HG to miR-16-5p.

**Results:**

MIR497HG was decreased in breast cancer and had high diagnostic function, while elevated MIR497HG inhibited the migration and invasion of BT549 and Hs 578T cells. In terms of functional mechanism, miR-16-5p was the target of MIR497HG, and MIR497HG reversely regulated the miR-16-5p. miR-16-5p mimic reversed the effects of upregulated MIR497HG on cell biological function.

**Conclusions:**

In general, MIR497HG was decreased in breast cancer, and the MIR497HG/miR-16-5p axis regulated breast cancer tumorigenesis, providing effective insights for the diagnosis of patients.

## Background

Breast cancer is called “pink killer”, and its incidence is usually located in the breast epithelium. The global cancer data claims that breast cancer has overtaken lung cancer as the cancer with the highest incidence rate in the world [1, 2]. The pathogenesis and specific etiology of breast cancer has not been fully reported, and there are multiple high-risk factors for the disease [3]. In addition, there are various types of breast cancer, so the treatment of patients is very difficult. The diagnosis of breast cancer is generally combined with the identification of breast mass and related pathological examination. Endocrine therapy, chemotherapy or targeted therapy are selected according to the clinicopathological conditions of patients, among which surgical resection is the first-line therapeutic schedule [4]. Factors such as patients’ health awareness, cancer prevention concept and medical conditions affect the diagnosis of breast cancer [5, 6]. Therefore, in addition to regular physical examination, the development and popularization of diagnosis and treatment methods are also critical.

Long noncoding RNAs (lncRNAs) regulate gene expression or protein transcription and processing in multiple links, which gradually become functional factors involved in the process of disease [7]. It has been reported that lncRNA carrying miRNA response elements has the effect of endogenous competition in theory [8]. Especially in the exploration of tumor mechanism, Wang stated that lncRNA RP11-551L14.4 binds to miR-4472 and regulates its level to control the production of breast cancer [9]. Fang et al. published that lncRNA HLA-F-AS1 regulates miR-21-3p/PEG3 axis to influence the progression of ovarian cancer [10].

MIR497HG is a potential and exploitable tumor suppressor gene on chromosome 17p13.1 [11]. In recent studies, MIR497HG was implicated in mediating disease and controlling cellular abilities in bladder cancer [12], glioma [13], and oral squamous cell carcinoma [14]. In particular, Zhang et al. proposed that 7 core lncRNAs, including MIR497HG may be closely related to the diagnosis and prognosis of breast cancer patients by integrating data [15]. On the basis of the existing discussion, we carefully screened the lncRNAs with abnormal expression related to breast cancer, and finally determined MIR497HG as the research target to deeply understand its biological information in the molecular mechanism of breast cancer.

In this study, the level of MIR497HG was verified in breast cancer samples and its correlation with the downstream target miR-16-5p was explored. Moreover, the mechanism of MIR497HG/miR-16-5p axis in breast cancer was explored, providing candidate markers for the diagnosis and treatment of patients.

## Methods

### Collection of patient specimens

The First People’s Hospital of Neijiang provided 96 patients with breast cancer and an equal number of breast cancer negative volunteers. Inclusion criteria: patients diagnosed with breast cancer by two specialists; patients had no complications or other major diseases; none of the patients had received intervention therapy such as radiotherapy and chemotherapy before the current resection. Exclusion criteria: patients had received breast cancer-related treatment; patients also suffered from hypertension, diabetes and other diseases; patients had no self-awareness or refused to participate in this study. This study was conducted under the supervision of the Ethics Committee of the First People’s Hospital of Neijiang (Approval No. 2021-21).

After the patients agreed to participate in the study and signed relevant documents, the breast cancer tumor tissues and non-cancer tissues removed during surgery were successively collected as experimental specimens, which were frozen in liquid nitrogen and stored in low temperature refrigerator. Between the mean value of MIR497HG expression in breast cancer tissues, 96 patients were composed of low-MIR497HG group (*n* = 49) and high-MIR497HG group (*n* = 47).

### Culture of breast cancer cells

All cell lines selected for the study were provided by American Type Culture Collection (ATCC; USA), including breast cancer cells BT549 (Bio-72,983), BT474 (Bio-132,967), T47D (Bio-103,891), Hs 578T (Bio-54,123) and non-malignant breast epithelial cell MCF10A (Bio-10,317). In Dulbecco’s Modified Eagle Medium (DMEM) medium (Corning, USA) with 10% fetal bovine serum (FBS; Thermo Fisher Scientific, USA) and 1% antibiotic/antimycotic (Thermo Fisher Scientific, USA), the above five cells were inoculated separately and incubated in 37 °C incubator (containing 5% CO_2_). When the cell density reached 80–90%, the cells were subcultured, and the third passage of cells was used for subsequent experiments.

### Construction of overexpressed MIR497HG cells

Overexpressed MIR497HG (pcDNA3.1-MIR497HG) plasmid was constructed using pcDNA3.1 (Promega, USA) and transfected into BT549 and Hs 578T cells with lipofectamine 3000 reagent (Invitrogen, USA).

### Detection of gene expression level

RNA from the samples was extracted by operation with TRIzol reagent (Invitrogen, USA), and the RNA concentration and purity was verified with a spectrophotometer (Thermo Fisher Scientific, USA), which should ensure that OD260/280 is between 1.8 and 2.0. GoScript Reverse Transcription Kit of Promega Company was purchased to reverse transcription RNA and prepare cDNA. The reaction system was configured with PrimeScript-RT Reagent Kit (Takara, Japan), while cDNA as the reaction template for RT-qPCR detection in Applied Biosystem 7900 system. glyceraldehyde-3-phosphate dehydrogenase (GAPDH) and RNU6 snRNA (U6) were endogenous controls for MIR497HG and miR-16-5p, respectively, based on their stable expression in most cells and independent of experimental conditions. The levels of genes were calculated by the 2^−ΔΔCt^ method. The primer sequences were listed as following: MIR497HG, F: 5’-GAGATCTCTTGTGGGGGTGC-3’ and R: 5’-ACGTAGCAGGGTGTTTCAGG-3’; miR-16-5p, F: 5’-GCAGCACGTAAATATTGGCG-3’ and R: 5’-GTGCAGGGTCCGAGGT-3’; GAPDH, F: 5’-TGTGTCCGTCGTGGATCTGA-3’ and R: 5’-CCTGCTTCACCACCTTCTTGA-3’; U6, F: 5’-CTCGCTTCGGCAGCACA-3’ and R: 5’-AACGCTTCACGAATTTGCGT-3’.

### Transwell analysis

Transfected BT549 and Hs 578T cells were adjusted to 3 × 10^5^ (cells/well) and cell solution was siphoned to the upper layer of the Transwell chamber (Corning, USA) where DMEM medium (Corning, USA) had been added. After 24 h of culture, the cells moved to the lower layer were scraped and counted under a microscope (Olympus, USA) to analyze the migration level, and the operation steps were repeated three times. Besides, with the participation of Matrigel (BD Biosciences, USA), the invasion ability of breast cancer cells can be evaluated through similar operations.

### Luciferase gene reporter assays

The targeting relationship between MIR497HG and miR-16-5p was predicted by ENCORI platform, and then further confirmed by luciferase gene reporter assay. The MIR497HG gene fragment was amplified and cloned into the pmirGLO luciferase report vector (Promega, USA) to form the MIR497HG wild-type (MIR497HG-WT) and mutant-type (MIR497HG-MUT) vector. Then, the vector and miR-16-5p mimic were co-transfected into BT549 and Hs 578T cells through lipofectamine 3000 agent (Invitrogen, USA), and mimic negative control (NC) was confirmed as the control group. Cell luciferase activity was evaluated by the dual-luciferase report detection system (Promega, Beijing).

### Statistical analysis

Data analysis was conducted using GraphPad version 9.0 and SPSS 20.0 software packages. The Shapiro-Wilk test was employed to assess the normality of the data distribution. The data obtained from the experiment were evaluated by Student’s t-test or analysis of variance (ANOVA) with Tukey’s post hoc test. ROC curve was established according to the levels of MIR497HG to analyze the sensitivity and specificity of MIR497HG expression in the diagnosis of breast cancer. Chi-square test was used to evaluate the correlation between MIR497HG and clinical indicators of breast cancer patients. Pearson correlation analysis explored the relationship between MIR497HG and miR-16-5p in tumor tissues. *P* value < 0.05 was considered meaningful.

## Results

### Expression and diagnostic significance of MIR497HG in breast cancer

**Fig. 1:**
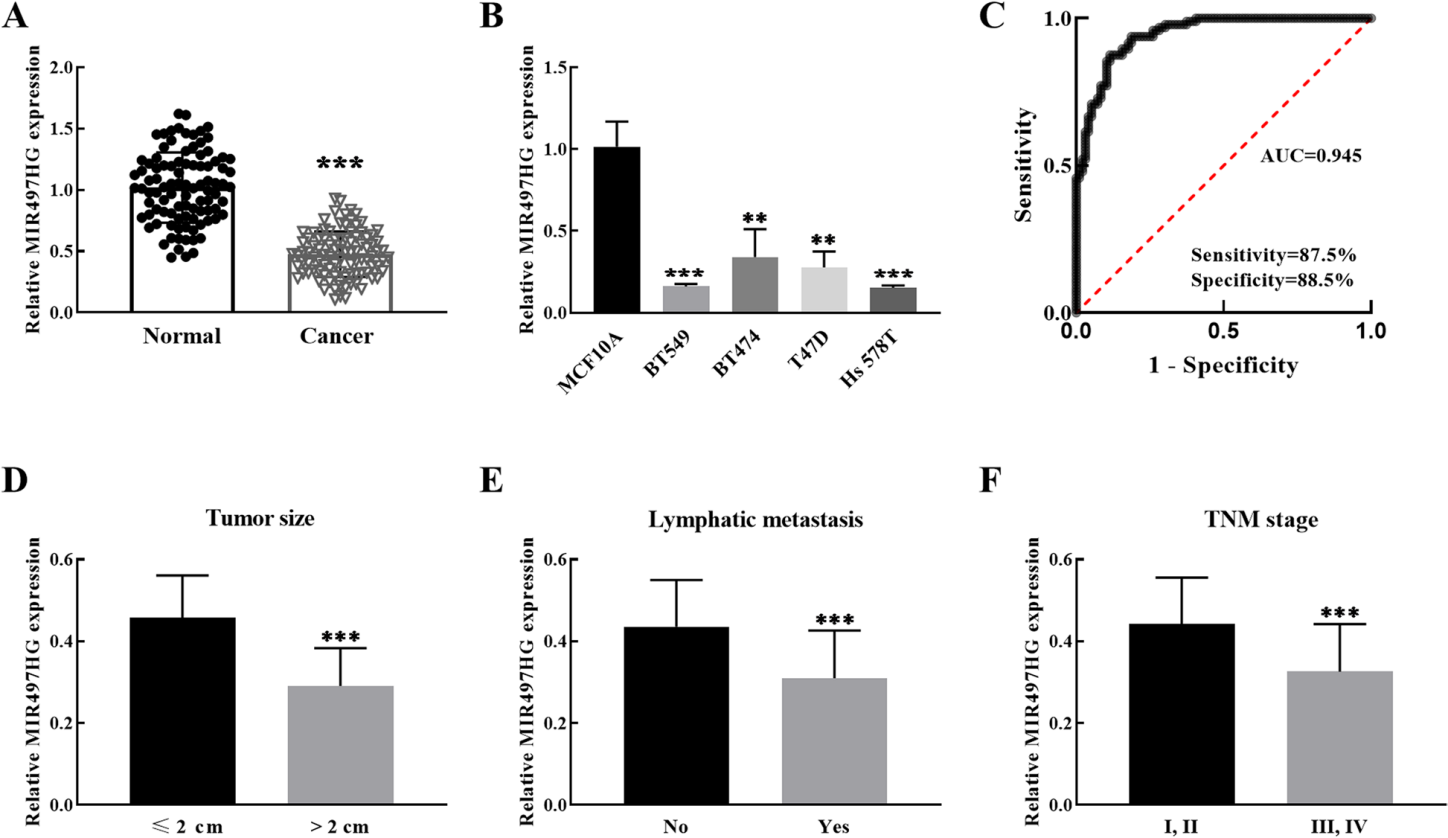
Expression and diagnostic function of MIR497HG in breast cancer. **A** MIR497HG expression in tumor tissue showed a decreasing trend. **B** MIR497HG decreased overtly in breast cancer cells. **C** ROC curve evaluated the AUC as 0.945. **D** MIR497HG expression in breast cancer tissues with tumor size. **E** MIR497HG expression in breast cancer tissues with lymphatic metastasis. **F** MIR497HG expression in breast cancer tissues with TNM stage. **P < 0.01, ***P < 0.001

MIR497HG level was detected by RT-qPCR, and it was remarkably reduced in cancer tissues compared with non-cancerous normal tissues, as shown in Fig. 1A. Moreover, MIR497HG expression in cultured BT549, BT474, T47D and Hs 578T cells was relatively lower than that in non-carcinogenic MCF10A cells (Fig. 1B). ROC curve was prepared according to the level of MIR497HG in breast cancer tissues. Figure 1C reveals that the area under the curve (AUC) of tumor samples and normal controls was 0.945 (Sensitivity = 87.5%, Specificity = 88.5%, 95% CI = 0.9164–0.9738), confirming the high diagnostic ability of MIR497HG for breast cancer.

### Analysis of clinical indicators of patients

In Table 1, Chi-square assay authenticated the association of MIR497HG expression with tumor size, lymphocytic metastasis and TNM stage (*P* = 0.021, *P* = 0.042 and *P* = 0.039) of breast cancer patients. Then, the relationship between relative MIR497HG expression and related pathological indicators was further analyzed. In Fig. 1D, tumor size was negatively correlated with MIR497HG expression. The larger the tumor size, the lower the MIR497HG expression. Figure 1E illustrates that lymphatic metastasis is associated with low expression of MIR497HG. Similarly, Fig. 1C implies decreased expression of MIR497HG at more advanced the tumor. The above means that the downregulated expression of MIR497HG may be the pathogenic gene of breast cancer.

**Table 1:** Relationship between MIR497HG and clinical indicators in breast cancer

Indicators	Low expression of MIR497HG	High expression of MIR497HG	*P*
*N* = 96	*n* = 49	*n* = 47	
Age			0.680
≤ 60	24	25	
> 60	25	22	
Tumor size			0.021
≤ 2 cm	28	38	
> 2 cm	21	9	
Distant metastases			0.195
No	34	38	
Yes	15	9	
Histological differentiation			0.307
Well, Moderate	22	26	
Poor	27	21	
Lymphatic metastasis			0.042
No	33	40	
Yes	16	7	
TNM stage			0.039
I, II	29	37	
III, IV	20	10	

### Function of breast cancer cells is regulated by MIR497HG expression

**Fig. 2:**
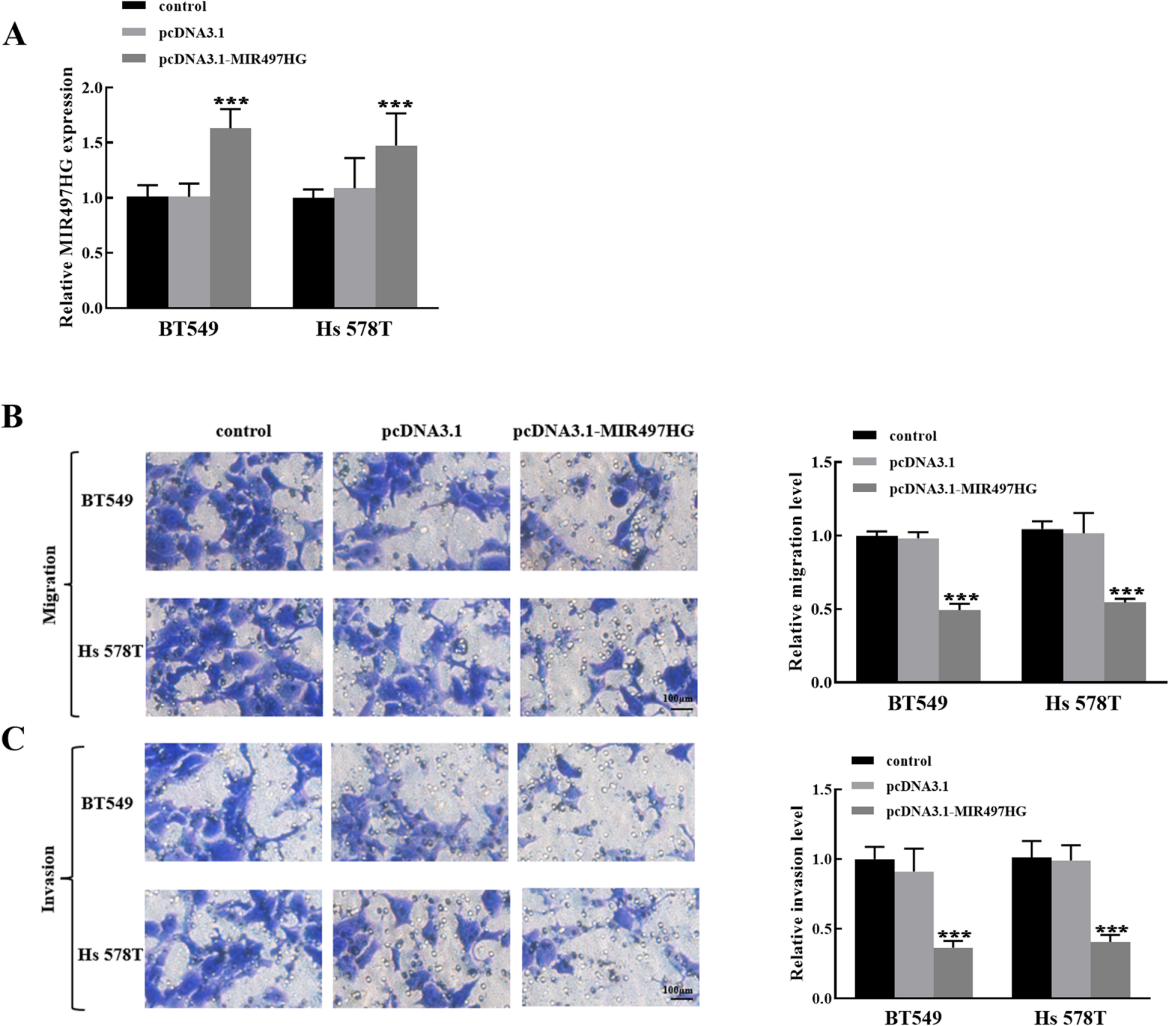
Effect of elevated MIR497HG on breast cancer cells. **A** Transfection efficiency of pcDNA3.1-MIR497HG was measured by relative MIR497HG expression in BT549 and Hs 578T cells. **B** Relative migration levels of BT549 and Hs 578T cells after transfection with pcDNA3.1-MIR497HG. **C** Relative invasion levels of BT549 and Hs 578T cells after transfection with pcDNA3.1-MIR497HG. ***P < 0.001

MIR497HG was overexpressed (pcDNA3.1-MIR497HG) in BT549 and Hs 578T cells, and the transfection results are shown in Fig. 2A. Furthermore, Transwell migration assay demonstrated that raised MIR497HG expression reduced the relative migration level of BT549 cells, and the same effect was observed in Hs 578T cells in Fig. 2B. Transwell invasion assay testified that pcDNA3.1-MIR497HG could reduce the invasion number of BT549 and Hs 578T cells (Fig. 2C). In conclusion, overexpression of MIR497HG blocked the biological activity of breast cancer cells.

### MIR497HG directly targets miR-16-5p

**Fig. 3:**
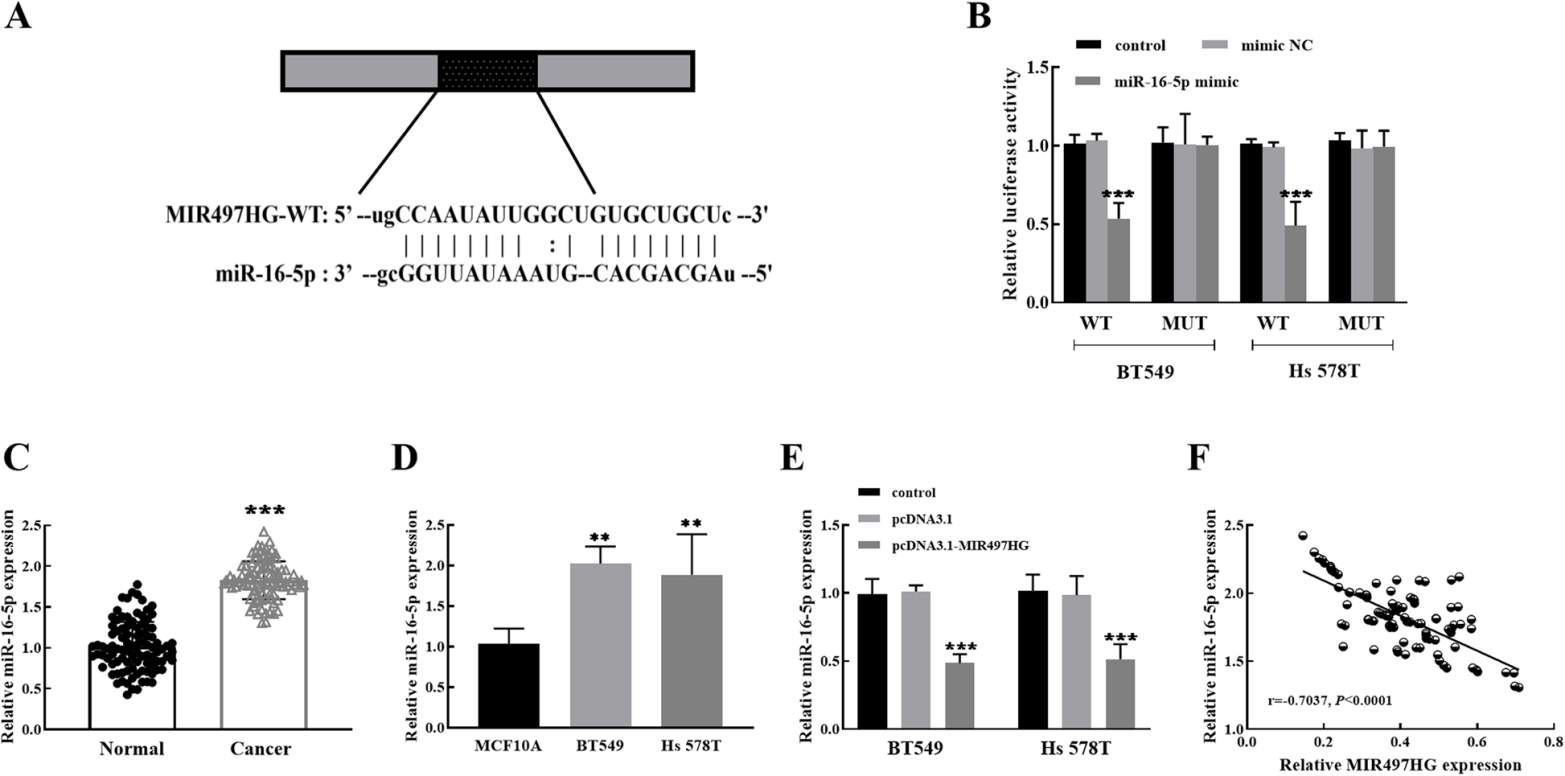
MIR497HG targets miR-16-5p to regulate breast cancer progression. **A** The binding sites existed between MIR497HG and miR-16-5p. **B** Luciferase activity was detected in BT549 and Hs 578T cells. **C** and **D** The upregulated expression of miR-16-5p was significant in breast cancer tissues and cells. **E** Relative miR-16-5p expression decreased after transfection of pcDNA3.1-MIR497HG in BT549 and Hs 578T cells. **F** There was a negative correlation between the MIR497HG and miR-16-5p expression in 96 breast cancer tissues (*r* = -0.7037, *P* < 0.0001). **P < 0.01, ***P < 0.001

Bioinformatics analysis revealed that the binding sites between MIR497HG and miR-16-5p, so we guessed that there was a potential relationship between them (Fig. 3A). In Fig. 3B, luciferase activity of BT549 or Hs 578T cells was reduced when miR-16-5p mimic was co-transfected with MIR497HG-WT, while transfection with MIR497HG-MUT had no significant effect. The results indicated that miR-16-5p was the direct target of MIR497HG. Subsequently, the enhanced miR-16-5p expression was verified by RT-qPCR in breast cancer tissue specimens (Fig. 3C) and cells (Fig. 3D). Additionally, pcDNA3.1-MIR497HG downregulated the miR-16-5p level in BT549 and Hs 578T cells in Fig. 3E. Spearman analysis assessed that the expression of MIR497HG was negatively correlated with the expression of miR-16-5p in breast cancer tissues (*r* = -0.7037, *P* < 0.0001), as demonstrated in Fig. 3F. Taken together, MIR497HG reversely regulates the downstream target miR-16-5p to affect the progress of breast cancer.

**Fig. 4:**
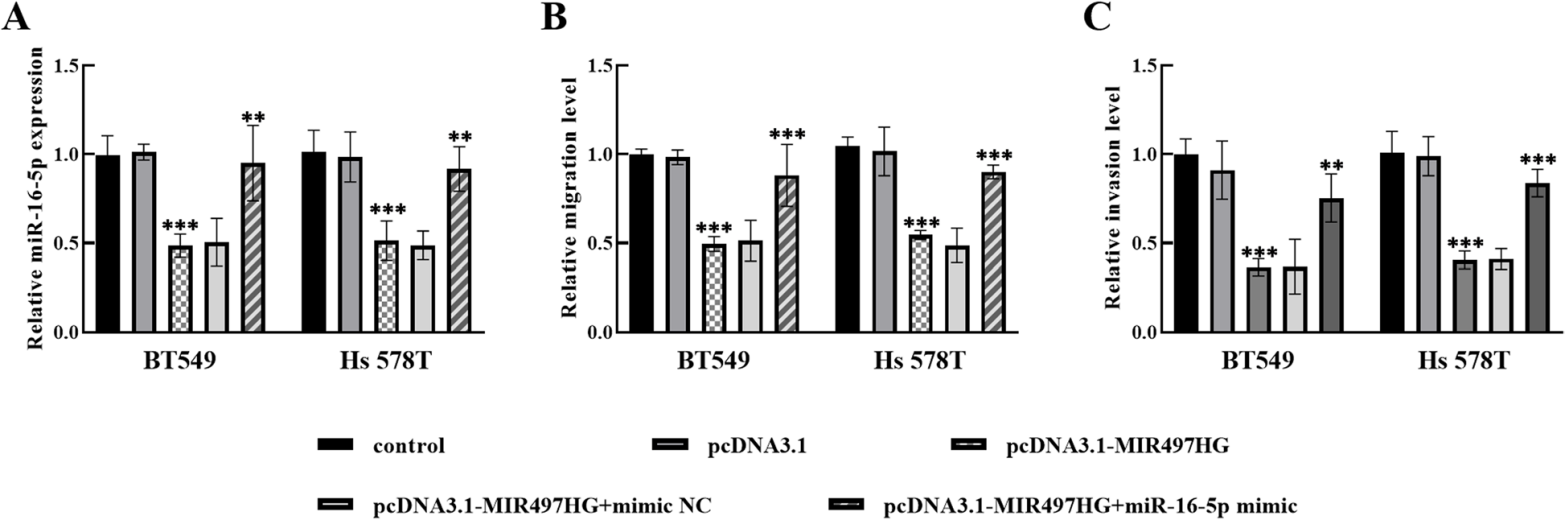
Regulation of cell biological functions by miR-16-5p mimic transfection. **A** Relative expression of miR-16-5p in cells after transfection with different indicators. **B**-**C** Elevated miR-16-5p reversed the biological function of overexpressed MIR497HG on breast cancer cells. **P < 0.01, ***P < 0.001

Moreover, transfection of pcDNA3.1-MIR497HG suppressed the miR-16-5p expression in BT549 and Hs 578T cells, whereas transfection of pcDNA3.1-MIR497HG + miR-16-5p mimic restored miR-16-5p expression, suggesting that co-transfection was successful in the cells (Fig. 4A). Transfection with miR-16-5p mimic counteracted the repressive effect of pcDNA3.1-MIR497HG on cell migration (Fig. 4B) and invasion (Fig. 4C).

## Discussion

With the increasing attention to the development of comprehensive treatment for breast cancer, the death toll of patients has declined in recent decades [16]. However, breast cancer is still the largest cancer afflicting women worldwide, with a negative situation [17, 18]. Currently, the differential expression of lncRNAs in tumors is often recognized to play a huge role in the prevention and treatment of diseases. For example, Xu et al. said that lncRNA and related miRNA could provide new train of thought for the study of anti-cancer targets in triple negative breast cancer (TNBC) [19]. Besides, lncRNA MALAT1 level has also been confirmed to be associated with lymphatic metastasis in breast cancer [20].

In our study, MIR497HG was observed to be negatively correlated with tumor size, lymphocyte metastasis, and TNM staging in breast cancer patients via Chi-square test, and downregulated expression of MIR497HG was evaluated by RT-qPCR. Meanwhile, ROC curve reflected the possible diagnostic significance of MIR497HG. Through literature review, lncRNA BC040587 was verified to be low expressed in breast cancer, and related to some clinical indicators of patients (age, grade, metastasis), with the potential of diagnostic and prognostic indicators of breast cancer [21]. LncRNA NEAT1 levels decreased, and the ability of NEAT1 to differentiate breast cancer patients was confirmed by multivariate analysis and ROC method [22]. The above findings were consistent with our experimental results. In the related description of MIR497HG, MIR497HG was downregulated in bladder cancer specimens, and markedly increased MIR497HG affected the normal growth of cells after construction. Therefore, Zhuang et al. speculated that MIR497HG played a non-negligible role in the malignant development of bladder cancer [12]. Tang et al. illustrated that MIR497HG was under-expressed, while MIR497HG overexpression suppressed the activity and progression of colorectal cancer cells [23]. Similarly, Transwell assay verified that the proliferation ability and migration level of BT549 and Hs 578T cells decreased when MIR497HG level increased in this study.

In the study of the pathological mechanism of breast cancer, it was found that MIR497HG may be the sponge of miR-16-5p, which was confirmed by the luciferase activity experiment. miR-16-5p has 22 nucleotides and is involved in the pathological processes of numerous diseases [24]. In the existing literature, the fact that miR-16-5p is bound by lncRNA in allergic rhinitis, osteoarthritis, cervical cancer, and colorectal cancer has been described [25–28]. Here, we highlight the elevated expression of miR-16-5p in breast cancer, and the inverse regulation of miR-16-5p by MIR497HG. However, transfection of miR-16-5p mimic counteracted the suppression of breast cancer cells by MIR497HG overexpression. In the discussion of the pathogenesis of preeclampsia, the authors stated that miR-16-5p was upregulated in the placenta of preeclampsia, and LINC00473 improved the symptoms of preeclampsia through sponge miR-16-5p [29]. MiR-16-5p was also identified as memorably enriched in Alzheimer’s disease and breast cancer, which is consistent with our results [30, 31]. Moreover, miR-16-5p was thought to contribute to the emergence of various tumors by directly targeting downstream ANLN, WEE1 and HMGA2, potentially serving as a candidate factor for patient therapy [32–34].

As for the mechanism study of MIR497HG/miR-16-5p axis in breast cancer, MIR497HG level was decreased, while the miR-16-5p expression was aggrandized. MIR497HG targets miR-16-5p and exerts negative regulation, and upregulated MIR497HG expression plays an inhibitory role in breast cancer. Unfortunately, there are still some shortcomings in this paper: (1) The limited sample size weakens the persuasiveness of the experimental results, and it is necessary to expand the sample size for additional research. By GPower calculation, when the effect size is set to 0.5, significant at 0.05, 172 participants are needed to achieve a statistical test power of 0.9. (2) The samples were from the same hospitals, which may be subject to selection bias and cause some error in the results. (3) Lack of relevant validation in animal models, and the molecular mechanism of MIR497HG with downstream miRNAs in breast cancer also needs to be supplemented in follow-up studies.

## Conclusions

In conclusion, MIR497HG/miR-16-5p axis is an action pathway that may regulate the progression of breast cancer, which brings new hope for the identification and treatment of patients.

## Data Availability

The datasets used and/or analysed during the current study are available from the corresponding author on reasonable request.

## Abbreviations

lncRNAs: Long noncoding RNAs
ROC: receiver operating characteristic curve
ANOVA: analysis of variance
